# Process Parameters Optimization of Rotary Friction Welding of Silicon Bronze CuSi_3_Fe_2_Zn_3_ Alloy Using Response Surface Methodology

**DOI:** 10.3390/ma19091877

**Published:** 2026-05-02

**Authors:** Henrique Pereira Machado, Francisco Yastami Nakamoto, Givanildo Alves dos Santos, Gilmar Ferreira Batalha, Vinicius Torres do Santos, Marcio Rodrigues da Silva, Flávia Gonçalves Lobo

**Affiliations:** 1 Department of Engineering, Federal Institute of Education, Science and Technology of São Paulo, São Paulo 01109-010, Brazil; nakamoto@ifsp.edu.br (F.Y.N.); givanildo@ifsp.edu.br (G.A.d.S.); 2 Polytechnic School, University of São Paulo, São Paulo 05508-010, Brazil; gfbatalh@usp.br; 3 Research and Development of Termomecanica São Paulo S.A., São Bernardo do Campo 09612-000, Brazil; vinicius.santos@termomecanica.com.br (V.T.d.S.); marcio.rodrigues@termomecanica.com.br (M.R.d.S.); flavia.lobo@termomecanica.com.br (F.G.L.)

**Keywords:** rotary friction welding, response surface methodology, silicon bronze, tensile strength, microstructure

## Abstract

This study investigates the optimization of selected process parameters in the rotary friction welding (RFW) process of CuSi_3_Fe_2_Zn_3_ silicon bronze alloys using Response Surface Methodology (RSM) with tensile strength as the primary response. The effects of rotation speed, friction time and friction pressure were evaluated, and the steepest ascent method was applied to determine the best parameters. The results indicated that rotation speed and friction time were the most influential parameters for enhancing tensile strength. A maximum tensile of 424 MPa was achieved under conditions of 3300 rpm, friction time of 25 s, friction pressure of 0.5 MPa, forging time of 16 s, and forging pressure of 8 MPa. However, confirmation experiments exhibited noticeable variability, indicating limitations in process repeatability. Tensile properties, hardness evaluation, microstructural characterization, and thermographic analysis were conducted to assess the quality of the welded joints. Microstructural analysis revealed recrystallized equiaxed grains in the welding center zone, consistent with severe plastic deformation, while microcracks and microvoids were observed and likely contributed for failure during tensile testing. Despite grain refinement, a reduction in microhardness was detected, suggesting the influence of thermal softening mechanisms. Thermographic analysis indicated that the average temperature at the welding center zone reached 564 °C. In conclusion, RSM proved to be a useful tool for identifying trends and guiding process optimization. The results highlight the importance of process stability and control in achieving consistent performance in RFW of copper-based alloys.

## 1. Introduction

Rotary friction welding (RFW) is a solid-state joining process in which the faces of two parts are placed in contact and under a certain pressure while undergoing relative motion. The heat generated by the friction softens both surfaces, and the flow of material starts the weld [1,2]. As it is widely used to manufacture components in a large number of applications, such as in aerospace, automotive and nuclear industries [3,4,5,6,7], factors such as durability, structural strength, low weight and corrosion resistance are essential requirements. Any structure in an engineering project requires the assembly of different components, and therefore the joining method is a key factor [8]. Considering this perspective, friction welding stands out as an alternative process for joining similar and dissimilar materials to obtain strong and reliable joints, as well as the advantage of low energy consumption, absence of toxic gases and vapors that harm the environment, relatively low labor skills and easy automation of the process [9].

The quality of the friction welding is directly influenced by a combination of design, material and process parameters [10]. Among these, process parameters such as rotational speed, friction time, friction pressure, forging time and forging pressure play a dominant role in controlling heat generation, material flow and microstructural evolution [2,3,11,12,13]. Inadequate parameter selection may result in insufficient bonding [13].

Several studies have investigated the influence of individual RFW parameters on joint performance. Abdul Ghani Khan et al. [14] studied the influence of rotational speed on the mechanical properties of LM25 cast aluminum alloy using RFW, and concluded that the highest rotation speed had a greater effect on grain morphology, due to the intense plastic deformation and dynamic recrystallization in the WCZ, compared to the lowest rpm. A reduction in ductility with increasing rotation speed was also observed. Li and Wang [15] investigated the effect of varying rotational speed on the temperature of the welding interface and axial reduction, considering a given axial pressure, concluding that the higher the speed, the greater the axial reduction in the same time interval, demonstrating a correlation between increasing rotational speed and increasing the rate of heat generation. Kumar et al. [16] used the Taguchi method to study the effects of RFW parameters on the joining of tubes in similar UNS S31803, keeping a constant speed of 1100 rpm, varying friction time and load in the friction and forging phases. Similarly, Kimura et al. [17] varied friction pressure, forging pressure and time, holding rotational speed at 1650 rpm, to investigate the effects of RFW on tensile strength involving pure copper and AISI 304 austenitic stainless steel. Studying the RFW parameters of the Bronze–Aluminum–Nickel alloy, Tchernov et al. [18] concluded that the friction time was the factor with the greatest contribution to the axial shortening of the workpieces. Additionally, Huber et al. [19] applied RFW to combine an E355 steel pipe with EN AW-6082 aluminum alloy and concluded that friction time was the most influent parameter to significant change the microstructure of the joint area, followed by rotational speed. Friction time was kept constant, rotational speed was adjusted by ±20% and friction pressure and forging pressure were adjusted by ±25%, from their base values.

While these works provide valuable insights, some of them focus on isolated parameter effects or limited parameter combinations. To address this limitation, a technique that has been used by different researchers [12,20,21,22,23,24] to analyze the correlation between the variables of the friction welding process in terms of their influence on the tensile strength and microhardness is the Response Surface Methodology (RSM), a collection of statistical and mathematical techniques used to develop, improve and optimize processes, which has extensive application in the industrial sector, particularly in situations where a selection of variables influences performance and quality characteristics of products and processes [25].

Sahin, Misirli and Selvi [21] applied RSM to optimize the joint between AISI 304 austenitic steel and aluminum, varying parameters based on friction pressure to friction time ratio, forging pressure to forging time ratio and rotation, in order to determine the maximum tensile strength. Friction pressure to friction time was identified as having a more significant effect on the tensile strength. Khalfallah et al. [12] used RSM to estimate the process parameters of rotary friction joining between AA1100 aluminum alloy and a low carbon steel to achieve optimum mechanical properties such as tensile strength and microhardness. A maximum strength was found to be under the maximum level of forging pressure/time. Belkahla et al. [20] adopted RSM to model the maximum bending strength of a rotary friction joint between C45 and 16NiCr6 steels by varying rotation, friction pressure and friction time, maintaining forging pressure and forging time as constant. Sundaraselvan et al. [24] joined Al 6082 T6 and Mild Steel workpieces in order to maximize tensile strength by applying RSM, varying friction and forging time, friction and forging pressure, and keeping the rotational speed as constant. Those authors concluded that friction pressure and forging pressure were the factors that most contributed to tensile strength. Selvaraj et al. [26] applied RSM to join SA 213 T12 and SA 213 F12 low-alloy steel tubes using rotary friction welding. By keeping friction and forging time constant, the authors found out that rotational speed had a significant impact on the tensile strength.

These studies are often focused on steels and aluminum alloys. In this context, a key gap remains in the literature regarding the comprehensive understanding of RFW applied to silicon bronze alloys, particularly CuSi_3_Fe_2_Zn_3_, a special alloy that meets international designation UNS C65620 and AMS4616 standard [27].

The addition of iron to copper alloys influences grain size and shape and promotes a significant improvement in wear resistance when added above 0.3% weight [28]. Zinc improves the mechanical properties of copper and offers solubility around 35% at 20 °C [29]. Studies carried out by Doostmohammadi and Moridshahi [30] revealed that the addition of silicon to copper–zinc alloys causes a refinement of the microstructure and an increase in hardness, improving machinability properties. Based on this characteristics, typical applications of C65620 are anti-friction bearing cages, bearing raceways, spacers, retainers, valve guides, gears, and hydraulic bushings, among others [27]. Despite its industrial relevance, no studies were found to understand the combined influence of process parameters, thermal history, and microstructural evolution on the mechanical performance of friction-welded joints in this material. Therefore, the objective of this study is to apply Response Surface Methodology to analyze and optimize selected process parameters in the rotary friction welding of CuSi_3_Fe_2_Zn_3_ silicon bronze, with a focus on tensile strength.

## 2. Materials and Methods

### 2.1. Material Selection

The base material used in this investigation was provided by Termomecanica São Paulo S.A. company [31]. Mechanical properties are as follow: minimum tensile strength is 386 MPa, minimum Yield Strength at 0.2% is 138 MPa, elongation in 4 times diameter is 30% and minimum Hardness is 90 HB/10/1000 (55 HRB). Chemical composition is presented in Table 1. Bars were produced by the wire drawing process and subsequent heat treatment, and were then cut into pieces 1 m long and with 5/8” nominal diameter.

**Figure 1:**
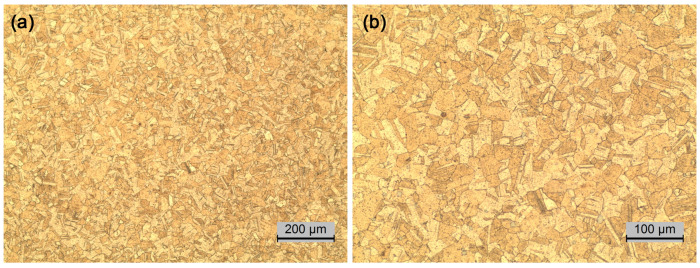
Optical micrographs showing the microstructure of C65620 sample: (**a**) 200×; (**b**) 100×.

Tensile tests were carried out; tensile strength (TS) average was 422.2 MPa, yield strength (YS) was 231.4 MPa at 0.2% and total elongation in 40 mm was 43.1%. Hardness tests carried out on the cross and longitudinal section indicated an average of 145.3 HB and 145 HB, respectively. The microhardness test showed an average of 180 HV in the cross section and 181 HV in the longitudinal section. Microstructure analysis revealed a polygonal grain shape in the cross section (Figure 1). Average grain size was found to be 0.025 mm.

### 2.2. Friction Welding Procedures

**Figure 2:**
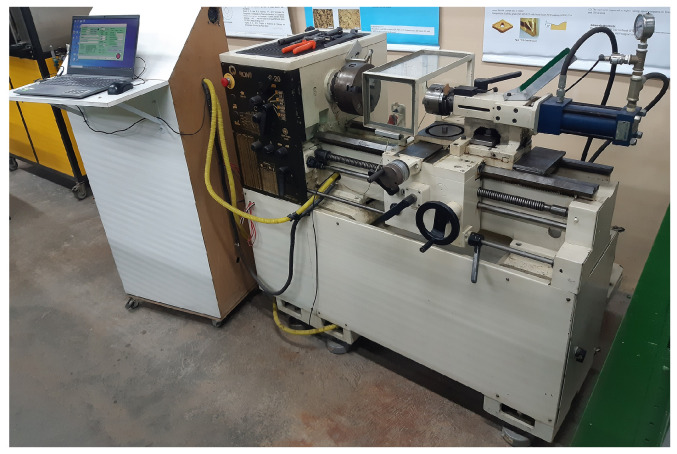
IFSP/SPO RFW experimental apparatus.

**Figure 3:**
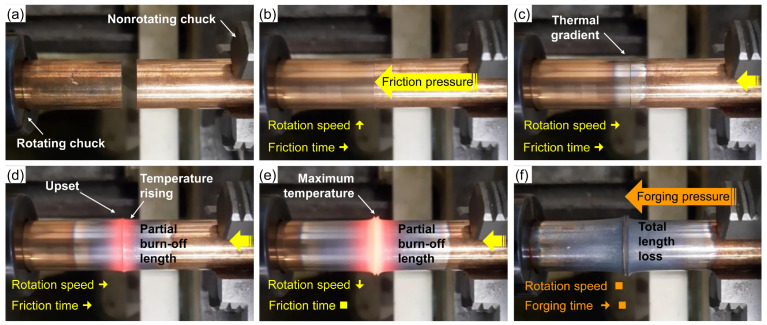
Process steps at the RFW experimental apparatus: (**a**) initial condition; (**b**) beginning of friction phase; (**c**) a few seconds after the beginning of friction phase; (**d**) intermediate friction phase; (**e**) end of friction phase; (**f**) forging phase.

The RFW experimental apparatus (Figure 2) from Federal Institute of Education, Science, and Technology of São Paulo (IFSP) was employed to develop the joints. As the access to large-scale machinery is limited, it consists of a conventional lathe adapted with an integrated hydraulic system by master’s degree students, along with modifications to the motor and transmission system. The instrumentation and implementation of the control system were developed through a collaborative effort involving graduate and undergraduate students [32]. A similar initiative can be found in [5]. Figure 3 illustrates the main steps of the welding procedure. The 5/8” nominal-diameter workpieces were shaped into 65 mm length. The following parameters were set at the control system: Rotation speed (*rpm*) [rpm], friction time (*t*1) [s], forging time (*t*2) [s], friction pressure (*P*1) [MPa], forging pressure (*P*2) [MPa].

### 2.3. Design of Experiments

The selection of the parameter ranges was based on a combination of preliminary experimental trials, equipment limitations, and literature data [18,33,34,35]. Initially, exploratory tests to evaluate the behavior of the silicon bronze CuSi_3_Fe_2_Zn_3_ alloy were conducted to identify feasible operating windows that ensured proper joint formation without excessive flash or premature failure. Previous studies reported that the factors most strongly influencing tensile strength and burn-off length are rotation speed, friction pressure, and friction time [16,18,20,21,23,36,37]. Accordingly, forging pressure and forging time were kept constant as 8 MPa and 16 s, respectively.

Response Surface Methodology (RSM) was applied to optimize the selected parameters to maximize the tensile strength (TS) of the welded joints. Total Length Loss (TLL) was also measured during the experiments. Accordingly, the number of factors *k* was set to three, and a Face-Centered Composite Design (FCCD) with two central points was employed. FCCD are effective for second-order models. Although not rotatable, they exhibit cubic symmetry. Moreover, only one or two center points are sufficient to ensure stable variance estimation [25].

Considering the FCCD design structure, the total number of experiments was calculated from Equation (1),

(1) $$N=2^{k}+2k+n_{c},$$

where *N* is the total number of runs, *k* is the number of factors and $n_{c}$ is the total number of center points. For this procedure, 2 center points were adopted, then *N* is equal to 16 experiments. Lower (−1), medium (0) and upper (+1) levels were assigned to perform RSM experiments, and the parameters values are listed in Table 2.

The ccd function, from the rsm package [38], available in the R program version 4.3.0 [39], installed on a Windows^®^ platform, with the integrated development environment RStudio 2023.03.1+446 was used to generate the design matrix, presented in Table 3. Output responses TLL, TS, YS and elongation are also presented in Table 3 and will be discussed in the next section.

Mathematical relationships between natural variables (ξ) and coded variables (*x*) are given in Equation (2).

(2) $$x_{i}=\frac{\xi_{i}-\left(\xi_{i}max+\xi_{i}min\right)÷2}{(\xi_{i}max-\xi_{i}min)÷2}\;i=1,2,\ldots ,k$$

Empirical relationship between the RFW parameters and TS response were estimated by a second-order polynomial regression model, as expressed in the form of Equation (3),

(3) $$y=\beta_{0}+\sum_{i=1}^{k}\beta_{i}x_{i}+\sum_{i=1}^{k}\beta_{ii}x_{i}^{2}+\sum_{i=1}^{k}\sum_{j=1}^{k}\beta_{ij}x_{i}x_{j}+\epsilon$$

where *y* is the output response, *β*_0_ represents the intercept, *β*_i_, *β*_ii_ and *β*_ij_ are the regression coefficients and ϵ represents the observed error. Model adequacy was checked using Minitab^®^ 18 and RStudio at a confidence level of 95%. Optimum parameters were determined by applying the method of steepest ascent provided by RSM [25]. The steepest function from the rsm package [38] was employed with each step varying by 0.1 sequentially up to 1.2, that is, slightly beyond the boundary of the design space (α = 1). Five experiments were carried out with the optimum parameters, and the tensile strength was analyzed.

### 2.4. Characterization Methods

Tensile tests were carried out on the welded joints in accordance with ABNT NBR ISO 6892-1:2024 [40] using a universal Instron 3385H tensile machine model, 250 kN load capacity and 20 mm/min crosshead speed [31]. According to AMS4616, four times (4D) nominal diameter (10 mm) was used to determine the elongation percentage.

Hardness tests were performed using Brinell scale in accordance with ABNT NBR ISO 6506-1:2019 [41] in a Wolpert Wilson Instruments 930/250N DigiTestor universal digital hardness tester, with a spherical 2.5 mm diameter penetrator, at 62.5 kgf load and 10 s application time [31]. Microhardness tests were recorded on a DiaTestor 2RC Wolpert durometer, at 1 N load and 15 s application time [31]. Vickers HV 0.1 scale was in accordance with ABNT NBR ISO 6507-1:2019 [42]. A Leica DM2700 M Upright Materials Microscope with Universal LED Illumination [31] was employed to observe the microstructure in accordance with ASTM standard E1245-03 [43].

**Figure 4:**
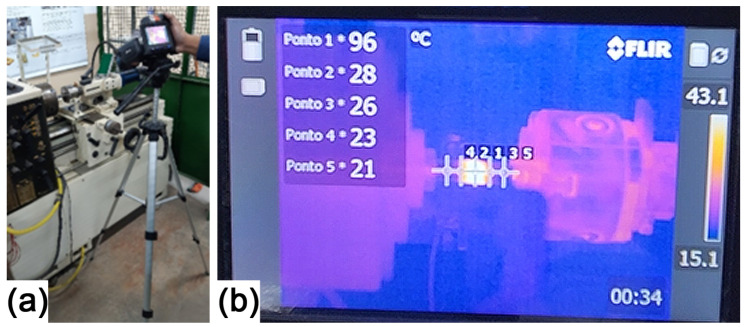
Temperature measurement procedure: (**a**) camera position; (**b**) thermographic camera model T650sc screen.

The FLIR model T650sc thermal imager was defined to perform temperature measurements. Temperature ranges are +100 to +650 °C and +300 to +2000 °C, image frequency of 30 Hz, accuracy of 2%, image resolution of 640 × 480 pixels and MPEG4 video recording. The camera was positioned on a tripod (Figure 4a) at a distance of 680 mm and 270 mm higher than the workpieces. The ambient temperature was set to 20 °C, relative humidity at 50% and emissivity at 0.82. Five measuring points were set up (Figure 4b): point 1 to Weld Center Zone (WCZ), points 2 (left) and 3 (right) approximately 10 mm far from WCZ, points 4 (left) and 5 (right) approximately 20 mm far from WCZ. Upper left frame on the thermal imager screen shows the continuous measurement condition of each point. For the first pair, a wide range (+300 to +2000 °C) was initially adopted, which prevented the detection of temperatures below 100 °C. Based on this result, subsequent trials employed a narrower range of 100 to 650 °C.

## 3. Results and Discussions

### 3.1. Friction Welding Experiments

**Figure 5:**
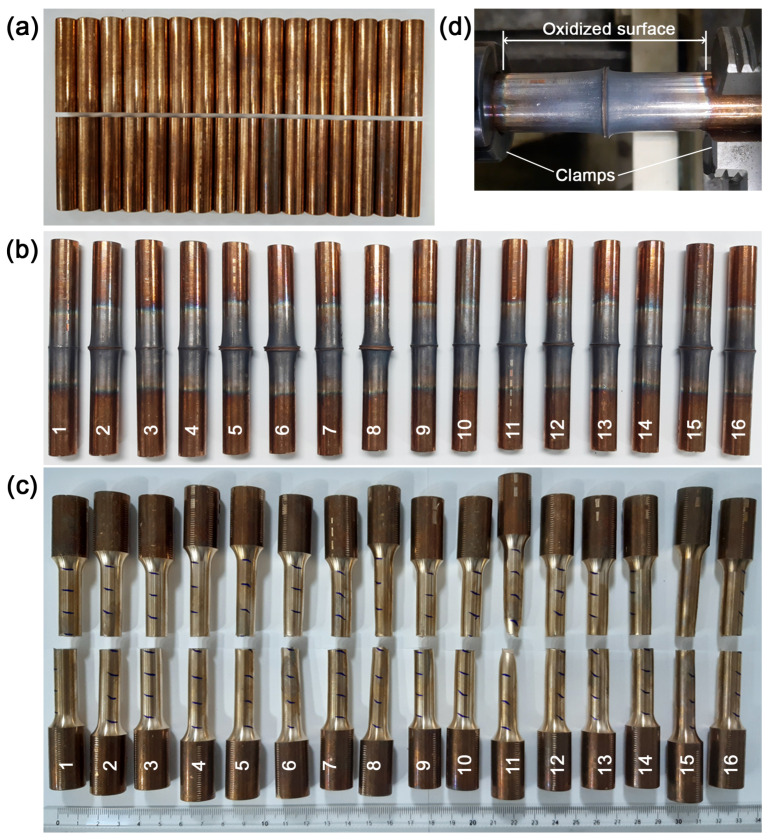
Macrographic views: (**a**) FCCD design matrix 32 workpieces; (**b**) 16 welded joints; (**c**) results of tensile tests; (**d**) oxidized surface appearance.

Macrographic views of the workpieces are presented in Figure 5: before welding (a), after welding (b) and after tensile testing (c). The thermal gradient extension, marked by a strongly oxidized surface, is shown in Figure 5d, indicating that heat generation was not confined to the immediate weld interface but extended along the axial direction. The clamping system partially constrained this effect, limiting oxidation along the longitudinal axis. This observation suggests that thermal dissipation and boundary conditions played a relevant role in defining the extent of thermally affected regions, which may have implications for both microstructural evolution and mechanical performance.

Table 3 summarizes the output responses. Total Length Loss (TLL) is calculated as the difference between the nominal (sum of the two workpieces’ lengths) and final lengths (after welding). Pair 1 failed at the onset of the tensile test and was still considered a valid response. The lowest TLL values occurred in Pairs 1, 14, and 3, all associated with shorter friction times. In contrast, higher rotation speed combined with longer friction time increased burn-off length, as observed in Pairs 6 and 8. The highest TS values were achieved in Pairs 4, 5, 6, 8, 11, and 15, all above 400 MPa, with an average of 408.7 MPa (3.2% below the base metal at 422.2 MPa). Notably, Pair 11 exhibited the best performance, being the only specimen to fracture outside the joining interface.

**Figure 6:**
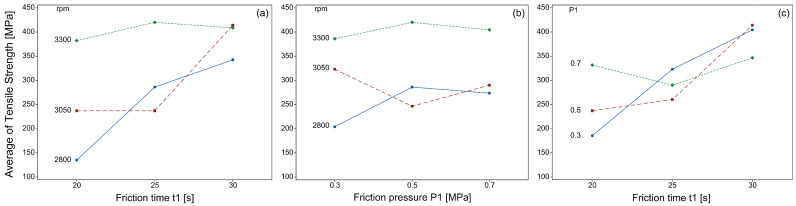
Interaction effects of the process parameters on tensile strength: (**a**) friction time and rotation speed; (**b**) friction pressure and rotation speed; (**c**) friction time and friction pressure.

According to Figure 6, both rotation speed and friction time exert a direct effect on tensile strength, as observed in Pairs 4, 5, 6, 8, 11 and 15. At lower levels of friction time and rotation speed, tensile strength decreased drastically. Moreover, it was observed that rotation speed required longer friction times to maximize tensile strength. A comparison between Pairs 6 and 8 reveals a difference of only 0.4 MPa in tensile strength, suggesting that friction pressure did not exert a significant impact in tensile strength. In contrast, when comparing Pairs 9 and 13, a considerable difference in tensile strength was observed despite a friction pressure variation of only 0.2 MPa. From Figure 6b, it is evident that the effect of friction pressure on tensile strength becomes pronounced only when rotation speed is at its higher level. Similar behavior is shown in Figure 6c with respect to friction time.

Pairs 9 and 16 correspond to central points in the experimental design and were therefore expected to yield similar tensile strength values. However, a significant discrepancy was observed, indicating limited process repeatability. All specimens were prepared using the same machining procedure to ensure consistent surface conditions. Factors such as surface roughness, alignment, and geometric tolerances were controlled as much as possible and kept constant throughout the experiments. This variability is likely associated with inherent limitations of the experimental setup, particularly related to hydraulic actuation stability and non-instantaneous braking. Such factors may introduce fluctuations in frictional heat generation and axial pressure application, directly affecting joint formation. This behavior highlights an important limitation of the present study, as part of the response variability may reflect machine-induced instability rather than intrinsic process behavior. Consequently, the predictive capability of the statistical model and the reliability of the identified optimum condition should be interpreted with caution.

Table 4 presents the estimated regression coefficients, whose significance was assessed using *p* values. Terms with *p* value < 0.05 are significant for the model, whereas terms with *p* > 0.05 are non-significant. Model adequacy (Equation (3)) was assessed by ANOVA (Table 5).

Simulations were conducted using first- and second-order models to achieve an optimal balance among the coefficients of multiple determination, residual normality assumption, and estimation error. Although the model was statistically significant (*p* = 0.027), its predictive capability remains limited, as indicated by the moderate R^2^ (73.69%) and relatively low adjusted R^2^ (56.15%). This discrepancy suggests that part of the variability is not adequately captured by the model, which may be attributed to experimental noise and process instability. Rotation speed (A) and friction time (C) were identified as the most influential parameters, whereas friction pressure (B) showed limited statistical significance. Nevertheless, factor B was retained to preserve model hierarchy, particularly due to its involvement in interaction terms [44]. It is important to emphasize that, given the observed variability, the model should be interpreted as a qualitative tool for trend identification rather than a precise predictive model.

**Figure 7:**
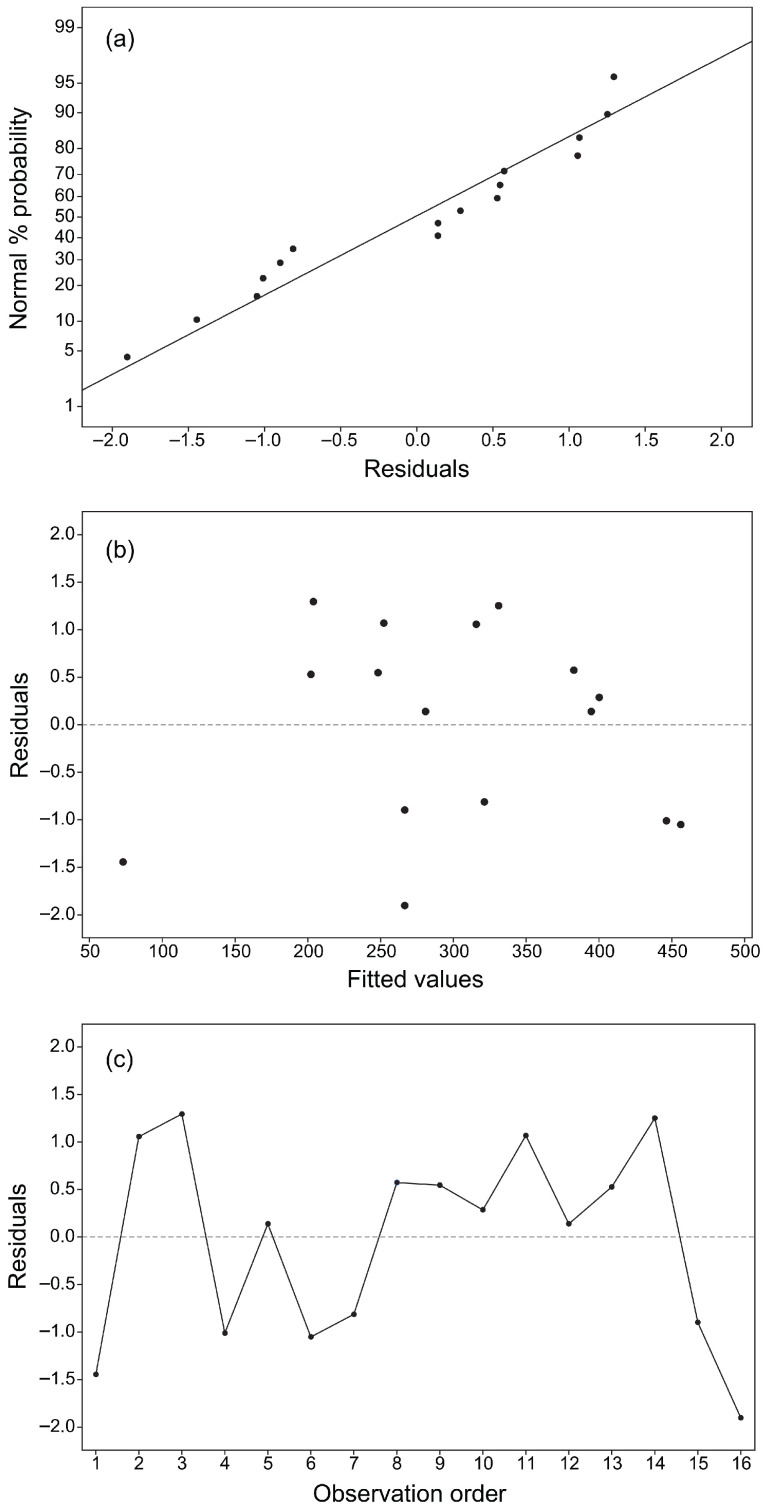
Plots: (**a**) normal % probability of residuals; (**b**) residuals versus fitted values; (**c**) residuals versus observation order.

The model of Equation (4), with the regression coefficients in Table 4, provided the best overall performance. Even if no substantial improvement in R^2^ and R_aj_^2^ was achieved, the standard error of all regression coefficients decreased. The lack of fit error was not significant, the normality assumption was satisfied (Figure 7a), and the residuals were randomly distributed (Figure 7b,c), confirming homoscedasticity of the tensile strength response.

Based on the estimated regression coefficients, Equation (4) represents the model in terms of coded values and Equation (5) in terms of actual factors:

(4) $$TS=266.5+76.02A+14.32B+64.52C-45.28AC-51.02BC+57.68A^{2}$$

(5) $$TS=4166-4.42rpm+1347P1+148.9t1-0.0362rpmt1-51P1t1+0.000923{rpm}^{2}$$

**Figure 8:**
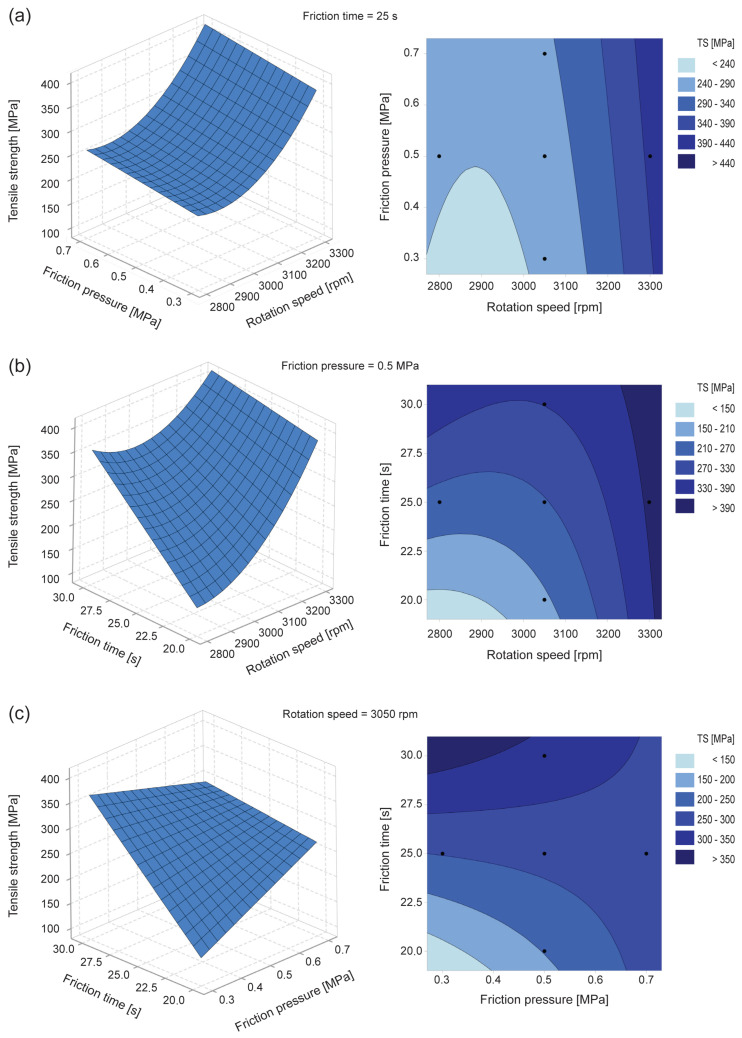
Response surface and contour plots on tensile strength: (**a**) friction pressure versus rotation speed at *t*1 = 25 s; (**b**) friction time versus rotation speed at *P*1 = 0.5 MPa; (**c**) friction time versus friction pressure at *rpm* = 3050 rpm.

Figure 8 shows the response surface and contour plots for tensile strength. Each plot depicts the interaction between two factors on the XY-plane while the third factor is held constant at its intermediate level; the surface response is represented on the Z-axis. The response surfaces indicate that tensile strength increases with rotation speed and friction time (Figure 8a,b). However, the presence of local minima and saddle points (Figure 8c), rather than a well-defined maximum, suggests that the response surface is strongly influenced by experimental variability. This behavior deviates from the expected monotonic trend typically observed in friction welding processes and reinforces the hypothesis that process instability affected the experimental outcomes. Therefore, the location of the stationary point at −0.5488360 (2912.8 rpm), 1.7514659 (0.85 MPa) and 0.2806467 (26.4 s), corresponding to a predicted TS point of 267.23 MPa (see Equation (4)), should not be interpreted as a true optimum, but rather as a mathematical aspect of the fitted model.

As these parameters do not maximize the response, RSM methodology provides the steepest ascent method, which consists of a step-by-step procedure along the path within the experimental region toward large response values, considering the central region as the starting point [25]. Using steepest function from the rsm package [38], the factor coordinates were sequentially varied from 0.1 to 1.2, slightly beyond the experimental domain (α = 1) in the ascending direction. Table 6 displays the outputs with A, B and C in coded units. The predicted response reached the target value at step 10 and increased by approximately 20 MPa in subsequent steps, accompanied by a rotation speed increment of about 25 rpm. In contrast, friction pressure and friction time exhibited no substantial variation. Considering equipment limitations, rotation speed was restricted to 3300 rpm, while friction pressure and friction time were fixed at 0.5 MPa and 25 s, respectively. These parameters correspond to experiment 11, the only case in which fracture occurred outside the bond interface.

**Figure 9:**
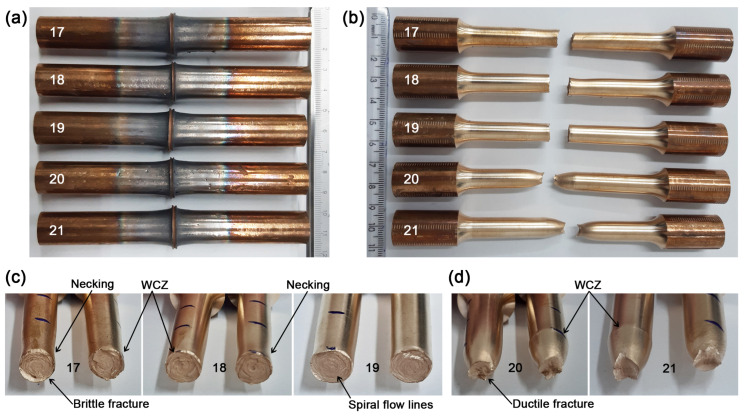
Macrographic views of validation experiments assigning optimized parameters *rpm* = 3300 rpm, *P*1 = 0.5 MPa and *t*1 = 25 s: (**a**) welded joints; (**b**) tensile tests results; (**c**) pairs 17, 18 and 19 showing brittle fracture at WCZ; (**d**) pairs 20 and 21 showing ductile fracture out of WCZ.

Five samples were produced for tensile testing under the optimized parameters. Table 7 summarizes the results. Figure 9 show the specimens after welding (a) and after tensile testing (b), respectively. The confirmation experiments exhibited noticeable scatter (381–424 MPa), despite identical nominal parameters. Although the mean value (402 MPa) is in good agreement with the predicted response (403.5 MPa) from Equation (5), the variability raises concerns regarding process robustness. Notably, two samples did not meet the AMS4616 minimum requirement, and distinct fracture modes were observed under the same conditions. This indicates that maximizing tensile strength alone may not be sufficient to define optimal parameters from a practical standpoint, and that process stability and repeatability should be considered as additional optimization criteria.

Pairs 17, 18, and 19 exhibited brittle behavior with a fracture occurring at the bond interface (Figure 9c). The presence of spiral flow lines is consistent with inertial rotary friction welding [45]. This behavior is likely associated with non-instantaneous braking of the system, as at the end of the friction time, the chuck requires additional time to come to a complete stop. Consequently, it suggests that the material cools while the chuck remains in motion. However, this interpretation remains speculative and should be considered with caution, as no direct measurement of deceleration was performed.

**Figure 10:**
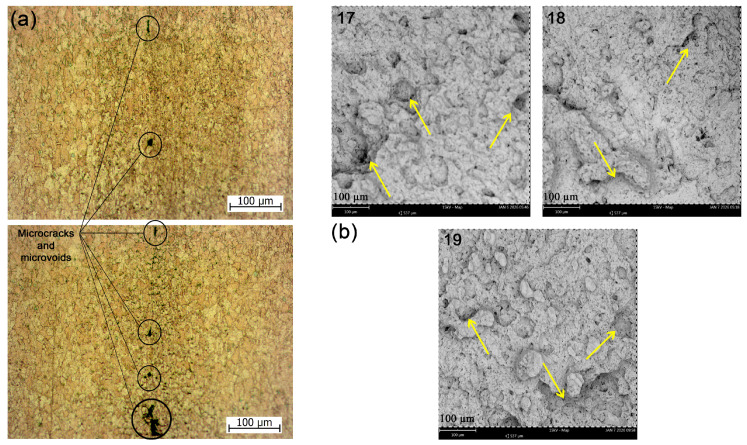
Micrographic views: (**a**) microcracks and microvoids at WCZ, Zeiss microscope, 200×; (**b**) dimples and microvoids at the fracture surface, Scanning Electron Microscope (SEM), (500×).

The presence of microcracks and microvoids observed on the weld surface (Figure 10a) may reduce structural integrity and is expected to have a detrimental effect on long-term durability and reliability, likely contributing to premature failure under load conditions. Nevertheless, the exact origin of these defects cannot be conclusively determined within the scope of the present study and may involve a combination of thermal gradients, material flow instability, and process control limitations. Figure 10b presents the fracture morphology of the 17, 18 and 19 pairs after tensile test at the center position of the diameter, revealing a highly irregular surface, indicating a dimpled intergranular fracture in all cases.

Pairs 20 and 21 showed ductile behavior with fracture outside the bond interface (Figure 9d), similar to pair 11. Tensile strength (383 and 381 MPa) were below the AMS4616 requirement of 386 MPa, despite identical parameters. One possible explanation is the influence of temperature on the extent of the Thermo-mechanically Affected Zone (TMAZ) and Heat-Affected Zone (HAZ), which may have induced microstructural changes leading to reduced strength. On the other hand, the differences observed in the results of pairs 17 to 21, as well as the variability noted in the previous 16 experiments, indicate the need for enhanced monitoring of the control system in the experimental apparatus in order to achieve finer control of the hydraulic actuators.

### 3.2. Hardness Analysis

**Figure 11:**
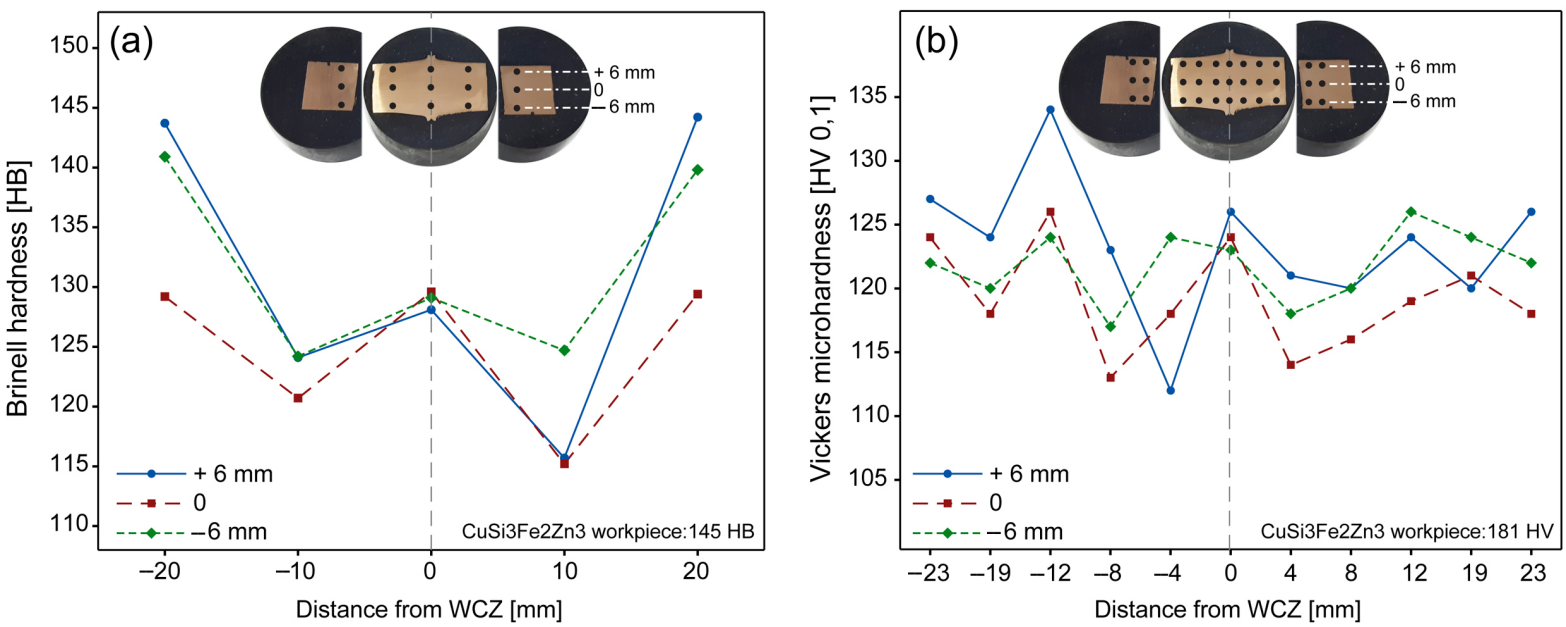
Plots for hardness tests: (**a**) Brinell hardness; (**b**) Vickers microhardness.

Brinell hardness tests (Figure 11a) showed values up to 129.6 HB in the WCZ, exceeding the minimum requirement AMS4616. However, Vickers microhardness results (Figure 11b) revealed a reduction in hardness despite grain refinement, which represents a non-trivial result. The base material exhibited 180 HV, while values decreased to approximately 125–134 HV after welding. This behavior suggests softening mechanisms may have outweighed the strengthening effect of grain refinement. It contrasts with previous studies reporting hardness increase in refined microstructures. Wei and Sun [46], for example, reported a nearly linear microhardness profile in copper prior to the welding interface with iron, along with an increase in microhardness within the contact region. Winiczenko [37] observed maximum microhardness values for both iron and steel near the contact interface. In Kannan et al. [36], a significant increase in microhardness was found for both AISI 1018 and AISI 1020 compared to the base metal region.

Possible explanations include thermal softening, microstructural homogenization, dynamic recrystallization and recovery processes leading to reduced dislocation density. This interpretation is consistent with the observed thermal gradients and highlights the complex interplay between thermal and mechanical effects during the welding process of this alloy. The application of post-welding heat treatments presents a promising avenue for future investigations.

### 3.3. Microstructure Analysis

**Figure 12:**
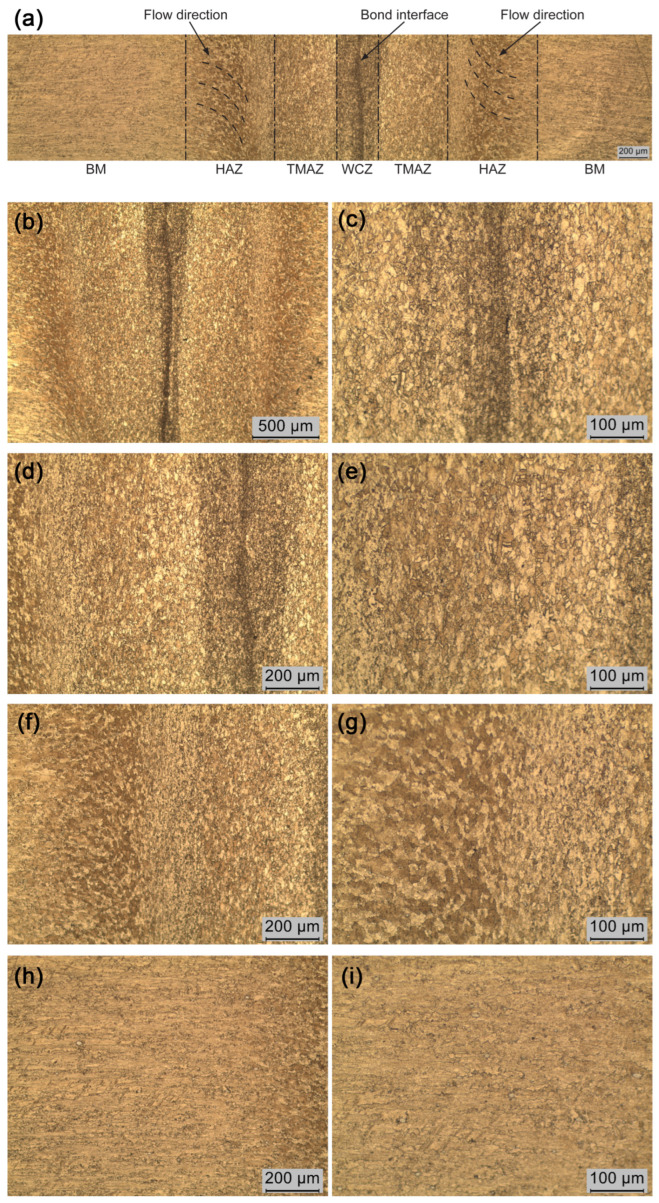
Micrographic views: (**a**) linear view compilation of the welding interface (200 µm, 50×); (**b**) WCZ 100×; (**c**) WCZ 200×; (**d**) TMAZ 100×; (**e**) TMAZ 200×; (**f**) HAZ 100×; (**g**) HAZ 200×; (**h**) BM 100×; (**i**) BM 200×.

A micrographic compilation is presented in Figure 12a. Figure 12b presents the micrographic view of the WCZ, where the joint line is visible. As reported in previous studies [11,47,48,49], the WCZ corresponds to a thin layer at the contact interface between the two surfaces. Due to severe plastic deformation, this region undergoes recrystallization and grain refinement [1,13,50]. In agreement with these findings, the present analysis revealed a recrystallized microstructure composed of equiaxed α-phase grains with an average size of 0.010 mm (Figure 12c). In contrast, elongated grains were observed toward the radial direction in regions subjected to axial compression during the friction and forging stages, indicating anisotropic behavior. The TMAZ also exhibited a recrystallized equiaxed α-phase grains, while the transition region between TMAZ and HAZ showed highly deformed, non-uniform microstructure. Figure 12f displays curved grain boundaries formed by the rapid cooling associated with simultaneous rotation and axial pressure during the final stage of welding. The base material (Figure 12h) displayed a microstructure lacking granular definition. At increasing distances from the WCZ, the microstructure was expected to resemble that of the original CuSi_3_Fe_2_Zn_3_ alloy (see Figure 1). However, complementary analyses indicated that this non-uniform morphology extended up to 23 mm from the weld interface, coinciding with the oxidized region (see Figure 5d). This indicates that the thermal gradient significantly influenced regions beyond the immediate weld zone.

### 3.4. Temperature Analysis

In light of the hypotheses established from the hardness and microstructural analyses, together with the pronounced surface oxidation observed during welding, surface temperature measurements were conducted. Although the thermal measurements were limited to surface temperatures, the results provide useful qualitative insights into the thermal cycle experienced during welding.

**Figure 13:**
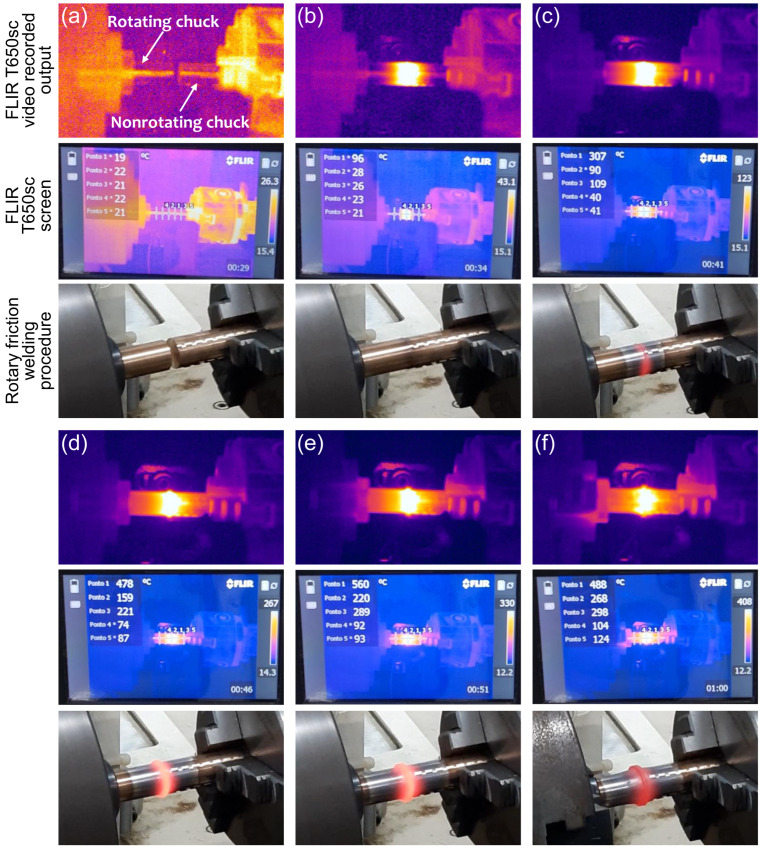
Thermal behavior stages, FLIR T650sc thermal camera: (**a**) initial condition; (**b**) beginning of friction phase; (**c**) a few seconds after the beginning of friction phase; (**d**) intermediate friction phase; (**e**) end of friction phase; (**f**) beginning of forging phase.

**Figure 14:**
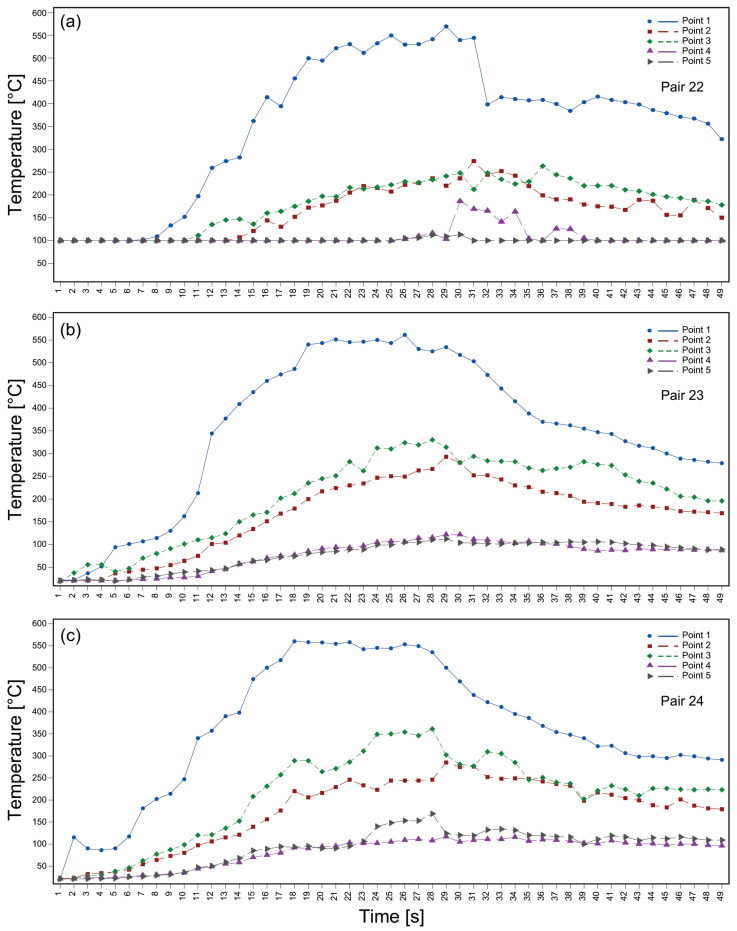
Temperature variation over time during welding procedure at the 5 measuring points: pairs (**a**) 22; (**b**) 23; (**c**) 24.

Thermal imaging using a FLIR T650sc camera revealed temperatures in the WCZ ranging 560 to 569 °C (Figure 13). The friction phase lasted approximately 3–28 s, during which a clear thermal gradient was observed. At its end, the WCZ reached 560 °C, while peripheral regions recorded temperatures around 124 °C. In the forging phase the rotational movement was interrupted and axial pressure was applied. The temperature evolution for a total duration of 49 s is plotted in Figure 14. Each data point corresponds to the temperature recorded at one-second intervals, covering the period from just before the friction phase up to the end of the forging phase.

According to [51], C65620 melting temperature is about 1100 °C. No specifications for annealing and hot working temperature ranges were found in the literature. However, specifications of other silicon bronze alloys such as C11000, C65100 and C65500, suggests that annealing temperature between 475 and 675 °C and hot working temperature between 700 and 875 °C are applicable to the C65620 alloy.

The temperature range observed in the WCZ is sufficiently high to promote dynamic recrystallization and microstructural transformation, which is consistent with the refined grain structure observed in this region. A significant thermal gradient was observed, with peripheral regions reaching 124 °C. This gradient likely contributed to heterogeneous microstructural evolution and may explain both the hardness reduction and the formation of defects. However, the use of infrared thermography restricts the analysis to surface temperatures, preventing accurate determination of subsurface thermal gradients. This limitation should be considered when correlating thermal history with mechanical and microstructural results. Overall, the results highlight the complex interplay between thermal effects, process parameters, and material response in rotary friction welding, as well as the importance of process stability for achieving reliable and reproducible joint properties.

## 4. Conclusions

This study investigated the optimization of selected process parameters in rotary friction welding of CuSi_3_Fe_2_Zn_3_ silicon bronze using Response Surface Methodology, with tensile strength as the primary response. The main findings can be summarized as follows:

Rotation speed and friction time were identified as the most influential parameters affecting tensile strength, whereas friction pressure showed a comparatively weaker effect within the investigated range. The statistical model developed was able to capture general trends; however, its predictive capability was limited, as indicated by the moderate R^2^ and adjusted R^2^ values.

The application of the steepest ascent method led to a set of process conditions (3300 rpm, 25 s, 0.5 MPa) associated with high tensile strength values. Experimental validation yielded a maximum tensile strength of 424 MPa, approaching that of the base material. Nevertheless, a noticeable scatter was observed in the confirmation tests, and not all samples met the AMS4616 minimum requirement, indicating that process robustness remains a critical issue.

Microstructural analysis revealed a recrystallized structure with equiaxed α-phase grains in the Weld Center Zone (WCZ) and Thermomechanically Affected Zone, consistent with severe plastic deformation and dynamic recrystallization. However, the presence of microcracks and microvoids in the WCZ suggests that local defects may have contributed to interfacial fracture under certain conditions.

Despite grain refinement, a reduction in microhardness was observed, indicating that softening mechanisms—such as thermal effects and recovery processes—may have outweighed the strengthening effect of reduced grain size. This behavior highlights the complex interaction between thermal cycles and microstructural evolution during RFW.

Thermal analysis indicated peak surface temperatures in the range of 560–569 °C at the WCZ, which are sufficient to promote microstructural transformations. However, the use of surface measurements limits the ability to fully characterize subsurface thermal gradients, and therefore restricts direct correlation with microstructural and mechanical responses.

Overall, the results demonstrate that, while RSM is a useful tool for identifying parameter trends and guiding process optimization, the presence of experimental variability—likely associated with limitations in the hydraulic control system and braking response—affects the reliability of the predicted optimum condition. Therefore, future work should focus on improving process control and incorporating robustness criteria into the optimization strategy, as well as on further investigation of thermal–microstructural–mechanical relationships in copper-based alloys.

## Figures and Tables

**Table 1:** Chemical composition (wt%) of C65620 [27].

Material	Cu	Si	Zn	Fe	Mn	P
CuSi_3_Fe_2_Zn_3_	min. 90.0	2.4–4.0	1.5–4.0	1.0–2.0	max. 1.0	max. 0.1

**Table 2:** Friction welding parameters for opmization experiments.

Parameter	Unit	Notation	−1	0	+1
(*rpm*)	rpm	A	2800	3050	3300
(*P*1)	MPa	B	0.3	0.5	0.7
(*t*1)	s	C	20	25	30
(*P*2)	MPa	D	8.0	8.0	8.0
(*t*2)	s	E	16	16	16

**Table 3:** FCCD design matrix and output responses.

Run Order	Std Order	Block	A	B	C	*rpm*	*P*1	*t*1	Pair	TLL [mm]	TS [MPa]	YS [MPa]	Elong. [%]
1	2	1	1	−1	−1	3300	0.3	20	2	2.36	364	185	11.50
2	3	1	−1	1	−1	2800	0.7	20	3	0.58	263	179	2.00
3	5	1	−1	−1	1	2800	0.3	30	5	3.36	401	164	20.17
4	8	1	1	1	1	3300	0.7	30	8	5.48	409	165	27.73
5	4	1	1	1	−1	3300	0.7	20	4	1.20	400	175	20.00
6	6	1	1	−1	1	3300	0.3	30	6	5.14	408	183	19.15
7	1	1	−1	−1	−1	2800	0.3	20	1	0.32	6.8	0	0
8	9	1	0	0	0	3050	0.5	25	9	0.98	203	139	3.48
9	7	1	−1	1	1	2800	0.7	30	7	1.32	284	192	4.85
1	4	2	0	1	0	3050	0.7	25	13	1.70	290	171	5.50
2	6	2	0	0	1	3050	0.5	30	15	2.92	414	189	25.70
3	1	2	−1	0	0	2800	0.5	25	10	0.80	286	191	3.95
4	5	2	0	0	−1	3050	0.5	20	14	0.52	237	189	2.83
5	2	2	1	0	0	3300	0.5	25	11	2.06	420	182	32.85
6	3	2	0	−1	0	3050	0.3	25	12	2.24	323	180	9.73
7	7	2	0	0	0	3050	0.5	25	16	1.40	132	105	1.65

**Table 4:** Estimated regression coefficients.

Factor	Coefficient	Standard Error	*t* Value	*p* Value
Intercept	266.50	31.62	8.43	0.000
*rpm*	76.02	24.49	3.10	0.013
*P*1	14.32	24.49	0.58	0.573
*t*1	64.52	24.49	2.63	0.027
rpm*t1	−45.28	27.38	−1.65	0.133
*P*1*t1	−51.02	27.38	−1.86	0.095
rpm*rpm	57.68	39.99	1.44	0.183

**Table 5:** ANOVA test results for the TS output response.

Source	Df	Sum of Squares	Mean Square	*F* Value	*p* Value
Model	6	151,173	25,195	4.20	0.027
Linear	3	101,469	33,823	5.64	0.019
Interaction	2	37,227	18,614	3.10	0.094
Quadratic	1	12,476	12,476	2.08	0.183
Residual	9	53,980	5998		
Lack of fit	8	51,459	6432	2.55	0.451
Pure error	1	2521	2521		
Total	15	205,152			
R squared	73.69%				
Adj. R squared	56.15%				

**Table 6:** Steepest ascent output.

Step	Distance	A	B	C	*rpm*	*P*1 [MPa]	*t*1 [s]	Response [MPa]
1	0.1	0.080	0.011	0.059	3070.00	0.5022	25.295	276.67
2	0.2	0.170	0.017	0.105	3092.50	0.5034	25.525	287.21
3	0.3	0.267	0.020	0.136	3116.75	0.5040	25.680	298.19
4	0.4	0.368	0.021	0.154	3142.00	0.5042	25.770	309.79
5	0.5	0.473	0.023	0.161	3168.25	0.5046	25.805	322.44
6	0.6	0.578	0.027	0.158	3194.50	0.5054	25.790	335.94
7	0.7	0.684	0.031	0.147	3221.00	0.5062	25.735	350.63
8	0.8	0.788	0.037	0.132	3247.00	0.5074	25.660	366.31
9	0.9	0.892	0.044	0.112	3273.00	0.5088	25.560	383.29
10	1.0	0.995	0.052	0.090	3298.75	0.5104	25.450	401.50
11	1.1	1.096	0.061	0.065	3324.00	0.5122	25.325	420.74
12	1.2	1.197	0.070	0.038	3349.25	0.5140	25.190	441.40

**Table 7:** Tensile strength responses of confirmation experiments: *rpm* = 3300 rpm, *P*1 = 0.5 MPa and *t*1 = 25 s.

Pair AB	TS [MPa]	YS 0.2% [MPa]	Elongation^40mm^ [%]	TL [mm]
17	424	181	33.63	3.88
18	409	190	27.10	3.16
19	413	190	25.00	3.54
20	383	166	31.25	2.42
21	381	175	29.85	2.92
Mean	402	180.40	29.37	3.18
Std deviation	19.08	10.26	3.40	0.56

## Data Availability

The original contributions presented in this study are included in the article. Further inquiries can be directed to the corresponding author.
